# 

**Published:** 1893-04-08

**Authors:** 


**OUR NEW OFFICES.**

428, Strand, W.C., will henceforth be the Office of this Journal.

### NOTICE TO CORRESPONDENTS.

All MS., Letters, Books for Review, and other matters intended for the Editor should be addressed The Editor, The Lodge, Porchester Square, London, W.

The Editor cannot undertake to return rejected M.S., even when accompanied by stamped directed envelope.

**Notice to Advertisers and Subscribers.**

All Advertisements, Orders for Copies of The Hospital, and Business Communications should be addressed to The Publisher, *not to the Editor*, The Hospital, 428, Strand, W.C.

The Cost of Subscription, post free, is as follows:—Per week, 2½d.; per quarter, 2s. 6d.; per half-year, 5s.; per annum, 8s. 6d. Foreign:—Per annum, 10s. 8d.; payable, in all cases, in advance.

**"Burdett's Hospital Annual," 1893.**

Fourth year of publication. 700 pp. Boards, gilt lettering. A most complete guide to British, Colonial, Indian, and American Hospitals, Dispensaries, Nursing and Convalescent Institutions. and Asylums. Price 5s. Published by The Scientific Press (Limited), 428, Strand, W.C.

**NOTICE.—The Index for Vol. XII. (ending September 1892) is on sale at the Office of this Journal, price 2½d., post free.**

### Scraps and Gleanings.

At a special convocation of Oxford University the degree of Doctor of Civil Law was conferred on Dr. Virschow.

The election of women on the Boards of Guardians is becoming more and more popular. Last year 140 were returned.

A curious ceremony took place lately in New Zealand, where a bride without arms had her wedding ring placed upon one of her toes.

Another hospital called "The Lebanon" has been added to New York. This establishment is situated in the Westchestor Avenue, 150th street.

The Lawn Hospital for the Insane, at Lincoln, has just received a legacy of £500 from the late John Julian, of Sleaford, a governor of the hospital.

The first meeting of the Society for the Protection of Birds was held last week in the offices belonging to the Society for the Prevention of Cruelty to Animals.

Some new eye-glasses have been invented by an American which, in combination with a small mirror, enables the wearer to see both in front and behind him.

At the meeting of the City Commissioners of Sewers at Guildhall, it was resolved to erect and maintain a public drinking fountain in front of St. Paul's Cathedral.

The City of London Parochial Charities have promised a donation of £500, in addition to the £1,000 already paid, for the acquisition of the Hilly Fields, Brockley, as an open space.

New sanitary regulations in Spain decree that all travellers entering the country must undergo a medical examination, and all rags, rotten fruit, and vegetables are prohibited entrance.

We hear from over the water that the wife of the American President is trying to bring a more simple way of hairdressing into fashion, and is waging quite a crusade against the universal fringe.

The twenty-fifth annual banquet of the French Hospital in London took place at the Hotel Metropole, M. Waddington being supported by the Lord Mayor; 250 persons were present, and a large sum of money was subscribed.

The Royal Infirmary, Glasgow, is to be congratulated on the success of its extended sphere of work. Besides reserving two wards for the exclusive use of women students, a scheme for instruction in nursing has been started.

Rochdale Infirmary reports that the new laundry and drying apparatus, also the large mortuary and offices which are in process of completion, have far exceeded the amount of the funds at the disposal of the institution.

The total number of persons treated as in-patients up to the end of last year at the General Hospital, Birmingham, was 206,158, and as out-patients 1,400,000. The average daily number of patients was computed at 233.

The Secretary of the British Home for Incurables has received a further donation of £250 from Mr. Pinter Simpson, thus completing a donation of £500, and founding a pension in connection with the institution to be known as the Mrs. Eliza Holm Pension.

Dr. Cameron has received a deputation at the House of Commons from the Federation of Grocers' Association to persuade him of the advisability of empowering inspectors to visit wholesale traders and manufacturers and take samples for investigation.

The Medical Board of the Bootle Borough Hospital have expresssed themselves ready to train volunteer nurses for any visitation of a cholera epidemic, provided that the Health Authority of the Borough guarantees to pay the nurses if called upon for duty.

The Committee for the Hostel of St. Luke, Devonshire Street, W., are making an urgent appeal to the public for £1,300. The institution is for the medical and surgical treatment and nursing of necessitous clergy and their families, either free or for a very small charge.

His Royal Highness the Duke of Connaught has graciously signified to Mr. Lennox Browne his pleasure to become patron of the Central London Throat and Ear Hospital, Gray's Inn Road, of which the Archbishop of Canterbury is president, and has headed the list of subscribers to the Building Extension Fund, rendered necessary by the increased demand for accommodation, by a donation of £20.

A pleasant entertainment was given to the patients and staff of the British Home for Incurables at Clapham on Wednesday evening through the kindness of Mr. W. F. Thomas, of the *Daily Graphic*, and Mrs. Thomas, Mr. and Mrs. Stratton, and Mr. and Mrs. Scott. Many ladies and gentlemen kindly gave their services. Miss Mabel Harrison, who gave a recitation, was presented with a bouquet by Mr. Salmond; the secretary, on behalf of the inmates of the Home.

For the Book World of Medicine and Science, and Student of Medicine, and Medical Appointments, see pages VIII., IX., X., and XI. overleaf.

### The Student of Medicine.

[All communications for this Supplement should be addressed to The Editor of The Hospital, at 428, Strand, with the words, "Students' Column," written in the left hand top corner of the envelope.]

### APPOINTMENTS.

Wm. A. H. Barrett, L.R.C.P.Lond., L.R.C.S.Edin., appointed Medical Officer of the Isolation Hospital, Guildford. H. Marten Body, M.R.C.S.Eng., reappointed Medical Officer for the Tedburn St. Mary District of the St. Thomas Union. George Henry Browne, L.R.C.P., L.M.Edin., L.F.P.S., reappointed Medical Officer of Health for the Brynmawr Urban Sanitary District. Francis John Butt, M.B., C.M.Edin., appointed Medical Officer for the North-Western Sanitary District of the Chester Union. Albert Carling, L.R.C.P.Lond., M.R.C.S., appointed Medical Officer to the Bristol Infirmary. Richard Clegg, L.R.C.P.Lond., M.R.C.S.Eng., appointed Medical Officer and Public Vaccinator for the District of Clayton-le-Moors and Rishton, Blackburn Union. F. J. Coleman, M.B., B.S.Lond., appointed House Physician to Guy's Hospital. A. M. Daldy, M.B., B.S.Lond., appointed Assistant House Physician to Guy's Hospital. William H. Date, M.R.C.S., appointed Medical Officer for the Third Sanitary District of the Wellington Union. Elliott Daunt, L.R.C.P.Lond., M.R.C.S.Eng., reappointed Medical Officer to the Brigg West District of the Brigg Union. Richard Domenichetti, Deputy - Surgeon - General, M.D., L.R.C.S.Edin., reappointed Medical Officer of Health for the Louth Rural Sanitary District. Thomas Jones Dyke, F.R.C.S.Eng., reappointed Medical Officer of Health for Merthyr Tydvil. C. E. Elliott, M.B., B.S.Lond., appointed Assistant House Physician to Guy's Hospital. James Anderson Goodfellow, M.B., C.M.Glasg., reappointed Medical Officer of Health to the Brampton Local Board. A. Goodridge, L.D.S., appointed Honorary Dentist to the Dartmouth Cottage Hospital. Henry Grace, L.R.C.P.Lond., M.R.C.S.Eng., reappointed Medical Officer of Health for the Kingswood Local Board. Charles Wm. Graham, L.R.C.P.Irel., appointed Medical Officer for the St. Cuthbert's Sanitary District of the Carlisle Union. Dr. Griffiths, appointed Medical Officer for the Western District of the Swansea Valley. B. Hall, M.B., C.M.Cantab., appointed Casualty Officer to the Leeds General Infirmary. E. T. E. Hamilton, M.B., B.S.Lond., F.R.C.S., appointed House Surgeon to Guy's Hospital. George James Harris, L.R.C.P.Lond., M.R.C.S.Eng., reappointed Medical Officer and Public Vaccinator of the Glenfield District of the Blaby Union. H. W. Haydon, M.R.C.S.Eng., appointed Medical Officer for the First District of the St. Colomb Major Union. William Andrew Hayes, M.R.C.S., L.R.C.P.Lond., appointed Medical Officer for the Second District of the Calne Union. Ernest Hill, L.R.C.P.Lond., M.R.C.S., appointed Second Assistant Medical Officer to the Leavesden Asylum, King's Langley. Silas Honeywill, L.R.C.P.Edin., M.R.C.S., appointed Medical Officer and Public Vaccinator of the Bildeston District of the Cosford Union, and Medical Officer to the Cosford Union Workhouse. E. Huntley, M.R.C.S., L.R.C.P., appointed House Surgeon to Guy's Hospital. Denis Kennedy, L.R.C.P., L.R.C.S.I., appointed Medical Officer of the Workhouse of the Stepney Union. Ernest E. B. Landon, M.R.C.S., L.R.C.P., appointed House Physician to Guy's Hospital. Philip Richard Littleton, M.R.C.S.Eng., reappointed Medical Officer of Health of the Ashbourne Urban Sanitary District. Donald Macaulay, M.A., M.B., C.M.Edin., appointed House Surgeon and Secretary to the Stamford, Rutland, and General Infirmary. Alexander A. Mackeith, M.B., C.M.Glasg., reappointed Medical Officer for the Brampford Speke District of the St. Thomas Union. Richard W. Marsden, M.B., Ch.B.Vict., M.R.C.S., L.R.C.P., appointed Assistant Medical Officer at the Crumpsall Workhouse of the Township of Manchester. J. T. Roger Miller, L.S.A.Lond., reappointed Medical Officer and Public Vaccinator for the Leadening District of the Malton Union. J. T. Neech, L.R.C.P.Edin., L.F.P.S.Glasg., reappointed Medical Officer of Health for Atherton. C. S. Pantin, M.B., B.S.Lond., appointed Assistant House Surgeon to Guy's Hospital. E. L. Puddicombe, L.R.C.P.Lond., M.R.C.S.Eng., reappointed Medical Officer for the Rewe District of the St. Thomas Union. F. M. Reynolds, M.B., C.M.Edin., appointed Medical Officer for the Fourth Sanitary District of the Honiton Union. F. H. Roberts, L.R.C.P.Lond., M.R.C.S.Eng., reappointed Medical Officer of Health for the Llandrindod Wells Urban Sanitary District. J. L. Roberts, M.D.Edin., D.P.H.Eng., reappointed Medical Officer of Health to the Abergele Local

### THE STUDENT OF MEDICINE—(continued).

Board. J. L. Russell, M.B.Edin., appointed Medical Officer for the Stansfield Sanitary District of the Todmorden Union. M. R. Saunders, L.R.C.P.Lond., appointed Medical Officer for the Hucknall Torkard District of the Basford Union. G. T. Sinclair, M.B., C.M.Edin., appointed by the Fife County Council Medical Inspector of Ships for Kincardine. W. St. Clair Symmers, M.B.Aberd., appointed Pathologist to the County Asylum, Prestwich, Manchester. T. R. Taylor, M.B., B.S.Lond., appointed Assistant House Surgeon to Guy's Hospital. Dr. Thomas, appointed Medical Officer of the Eastern District of the Swansea Valley. Walter Thompson, F.R.C.S.Eng., appointed Resident Surgical Officer to the Leeds General Infirmary. William E. Tressider, M.B., B.S.Lond., appointed Honorary Assistant-Surgeon to the Nottingham Hospital for Women. Edmond F. Trevelyan, B.Sc., M.D.Lond., M.R.C.S., appointed Pathological Curator to the Leeds General Infirmary. A. H. Tubby, M.S.Lond., F.R.C.S.Eng., appointed Surgeon to Out-patients, Evelina Hospital for Sick Children, Southwark Bridge Road, S.E. F. Webb, L.R.C.P.Lond., M.R.C.S., appointed Honorary Surgeon to the Doncaster Infirmary and Dispensary. Alex. H. Whicher, L.R.C.P., M.R.C.S., appointed Medical Officer of Health to the Midsomer Norton Local Board. Arthur Longley Whitehead, M.B., L.R.C.P.Lond., M.R.C.S., appointed Resident Ophthalmic Officer to the Leeds General Infirmary. J. Mackie Whyte, M.A., M.B.Edin., appointed Physician to the Dundee Royal Infirmary. Henry Williams, M.R.C.S., L.R.C.P., appointed Honorary Surgeon to the Nottingham Hospital for Women. John Humphrey Williams, M.D.Edin., appointed Medical Officer for the Flint District of the Holywell Union. W. G. Willoughby, M.D.Lond., D.P.H.Camb., reappointed Medical Officer of Health for Plympton.

### VACANCIES.

The following vacancies are announced this week, the amount of salary in each case being given after the name of the institution:

#### Resident Medical Officers.

County Asylum, Rainhill, near Liverpool. Assistant Medical Officer, unmarried, and not more than 30 years of age. Salary to commence at £100 per annum, with prospect of £25 increase at the end of first and second years, and with further increase according to promotion, together with furnished apartments, board, and attendance. Applications to the Medical Superintendent.

Flintshire Dispensary, Bagills Street, Holywell. Resident House Surgeon. Salary £120 per annum, with furnished house, rent, and taxes free, also coal, light, water, and cleaning, or in lieu thereof £20 per annum. Applications to William Thos. Cole, Secretary, by April 11th.

General Infirmary at Gloucester and the Gloucestershire Eye Institution. House Surgeon, doubly qualified. Salary £100 per annum, with board, lodging, and washing. Applications to the Secretary by April 12th.

Miller Hospital and Royal Kent Dispensary, Greenwich Road, S.E. Junior Resident Medical Officer. Salary £30 per annum, with board, attendance, and washing. Applications to the Honorary Secretary by April 15th.

Parish of St. Leonard, Shoreditch. Second Assistant Medical Officer for the Infirmary; doubly qualified. Salary £4[illegible] per annum, with rations, furnished apartments, and washing. Applications to the Medical Officer, 204, Hoxton Street, N.

Pontefract General Dispensary and Accident Ward, Pontefract. Medical Officer; doubly qualified. Salary £130 per annum, with furnished rooms, fire, lights, and attendance. Applications to Percy P. Wood, Secretary, by April 7th.

Poplar and Stepney Sick Asylum District, Bromley, Middlesex, E. Assistant Medical Officer. Salary £130 per annum, with rations, furnished apartments, and washing. Applications, on forms to be obtained at the Clerk's Office, to Robert Foskett, Clerk to the Managers, by April 10th.

Radcliffe Infirmary, Oxford. House Surgeon; doubly qualified. Salary £80 per annum, with board, lodging, and washing. Applications, on forms to be obtained of the Secretary, by April 7th.

Royal Free Hospital, Gray's Inn Road. Senior Resident Medical Officer; doubly qualified. Salary £100 per annum, with board, residence, and washing; also Junior Resident Medical Officer. Board, residence, and washing provided. Applications to Conrad W. Thies, Secretary, by April 17th.

#### Non-Resident Medical Officers.

City of London Hospital for Diseases of the Chest, Victoria Park, E. Pathologist. Salary 100 guineas per annum. Applications to the Secretary, at the office, 24, Finsbury Circus, E.C., by April 20th.

Dental Hospital of London, Leicester Square. Assistant Anæsthetist. Applications to J. Francis Pink, Secretary, by April 10th.

Hospital for Consumption and Diseases of the Chest, Brompton. House Physicians. Applications to the Secretary by April 13th.

Mason College, Birmingham. Co-Professor of Surgery. Applications to G. H. Morley, by April 15th.

St. Bartholomew's Hospital, Physician. Applications to Wm. Henry Cross, Clerk, by April 11th.

West Norfolk and Lynn Hospital, Lynn. House-Surgeon and Secretary. Salary £80, rising to £100 per annum. Applications to the Chairman of the Weekly Board by April 7th.

